# St. Bartholomew’s Hospital

**Published:** 1852-07

**Authors:** 


### PATHOLOGY AND PRACTICE OF MEDICINE.

St. Bartholomew's Hospital.—*Aneurism by Anastomosis; Death by Chloroform.* (Under the care of Mr. Lloyd.)—We regret to apprise our readers that the use of chloroform has proved fatal in this case. It will be clearly shown by the subjoined facts that no blame whatever can attach to any one concerned, the more so as the patient had, on the previous occasion, inhaled chloroform during a considerable time without any ill effects. We refrain from any comments, as we shall have occasion to return to the case when the post-mortem examination shall have been made.

Thomas Hayward, a rather spare and weakly man, aged twenty three, was admitted on Thursday, the 29th of January, 1852, under the charge of Mr. Lloyd.

The patient applied to the hospital for an aneurism by anastomosis occupying the whole of the right ear, and also to a considerable extent the soft parts in front and behind that organ. On and behind the ear, the vascular growth was elevated so as to form a large tumor. The integuments of the ear, as well as the diseased mass, were of a deep purple color. In every part there was a strong pulsation, as well as a loud aneurismal murmur; the temperature, however, was much the same as that of the surrounding parts. Projecting from the meatus there was a polypus or large fungus, whence issued a copious purulent discharge, which was often tinged with blood. Pain in the head was complained of, but no unpleasant sensations had been felt in the tumor itself.

The disease had existed since the patient was four years of age; various remedial means had been employed at different times, but without benefit. The patient had been treated at some of the London hospitals, where setons were introduced into different parts of the tumor, and portions of it enclosed with silver wires, which were now and then twisted so as to tighten them; but these measures were of no avail, and at length the patient was discharged as incurable.

Mr. Lloyd, not deeming the cure hopeless, and having consulted with his colleagues, determined to attempt the obliteration of the tumor—first, by deligation of the principal arterial branches in direct communication with the diseased mass, and afterwards by pressure applied to different parts in succession. With this view, on the 14th of February, 1852, the patient was placed under the influence of chloroform, and with the assistance of Messrs. Wormald and Paget, Mr. Lloyd placed a ligature around the temporal artery, just as it passes over the zygoma; other ligatures were applied in such situations as it was considered would tend most to cut off the supply of blood from the part affected, and pressure afterwards used before and behind the ear. The operation lasted, as was expected, for a long time, and the patient was kept under the influence of chloroform for *half an hour or more.* From the effect of the anæsthetic agent he recovered quickly, and when visited half an hour afterwards, he was found lying comfortably in his bed, and on being asked how he was, he answered, "Very well," and smilingly added that he was very thankful the operation had been performed, and hoped it could soon be repeated.

After this, everything went on favorably: the tumor became much diminished in size, and the pulsation much lessened. But on further examination, a large artery was found beating very strongly, between the mastoid process and the ramus of the jaw, pressure on which part completely arrested the pulsation throughout the diseased growth. On this vessel, therefore, Mr. Lloyd determined to place a ligature, and appointed the 17th of March for the purpose.

The patient was taken into the theatre of the hospital, placed on the operating-table, and chloroform administered as on the former occasion. The anæsthetic fluid now used was from the same bottle as had been employed before, and the apparatus was also the same as usual. The chloroform was administered by one of Mr. Lloyd's dressers, who well understood, and had long had experience in its use. A gentleman of great experience, who has been a long time at the hospital, and two years house-surgeon, was watching the patient, and marking the state of the pulse. Other gentlemen were also assisting.

In from five to ten minutes the usual effect was produced, the patient having previously struggled much. The operation was then commenced, but no sooner had Mr. Lloyd cut through the skin, than it was stated that the pulse had suddenly stopped.

The chloroform was at once removed, but in a few seconds the patient had ceased to breathe, and no pulsation could be felt in any of the arteries, or at the heart. Artificial respiration, as well as percussion and compression of the different parts of the body, were immediately employed, with energy; and after continuing the means for a short time, the circulation was observed to be returning, and the act of respiration was several times performed. The state of inanimation, however, speedily returned; but by the employment of the same means as before, with the use also of galvanism, the circulation and respiration were again restored. But the patient fell quickly into the same state as at first, and was again brought round by the same means. In a few moments, the patient relapsed for the third time, when one of Mr. Lloyd's colleagues, coming into the theatre, recommended that the external jugular vein, which on the right side was turgid, should be opened, that tracheotomy should be performed, and the lungs inflated. These means were accordingly had recourse to.

The patient was besides placed in a warm bath at the temperture of 104°, artificial respiration being kept up all the time, and friction employed. All, however, was of no avail, and it soon became evident that life was irrecoverably gone.

The resuscitating measures had been continued for more than an hour. Ammonia had been applied to the nostrils, but no attempt was made to introduce any stimuli into the stomach, as Mr. Lloyd feared any liquid placed in the mouth might pass into the larynx, and occasion instant suffocation.—*Lancet, March 20th,* 1852.

